# The impact of the SARS-CoV-2-pandemic on patients with chronic inflammatory neuropathies: results from the German INHIBIT register

**DOI:** 10.1007/s00415-022-11527-8

**Published:** 2022-12-22

**Authors:** Alina Hieke, Marie Spenner, Fynn Schmitz, Aurelian Schumacher, Maximilian Schröder, Rafael Klimas, Melissa Sgodzai, Jil Brünger, Thomas Grüter, Ralf Gold, Kalliopi Pitarokoili, Anna Lena Fisse, Jeremias Motte

**Affiliations:** 1 Department of Neurology, St. Josef-Hospital, Ruhr-University Bochum, Gudrunstr. 56, 44791 Bochum, Germany; 2 Immune-Mediated Neuropathies Biobank (INHIBIT), Ruhr-University Bochum, Bochum, Germany

**Keywords:** CIDP, COVID-19, Health care, Register, Vaccination

## Abstract

**Introduction:**

SARS-CoV-2 pandemic is especially compromising for patients with autoimmune diseases with or without immunomodulatory treatment. This study aimed to investigate the longitudinal changes in the health care of patients with immune-mediated neuropathies during the COVID-19 pandemic.

**Methods:**

We performed a longitudinal study using questionnaires in a prospective cohort of patients with immune-mediated neuropathies at two timepoints of the pandemic: May–July 2021 and May–July 2022.

**Results:**

The cohort consisted of 73 patients (55 male), mean age 62 years, 68 patients with CIDP, 5 with other immune neuropathies. In 2021, 19.2% of the patients reported a reduced number of physician–patient-contacts, while 13.7% reported this in 2022. Nevertheless, the overall health-care situation worsened from 2021 to 2022: 15.1% reported reduced overall healthcare in 2021, 26.0% in 2022. In 2021, 29.4% of patients reported absence of physio-/occupational therapy, while 34.4% reported this in 2022. Switching immunomodulatory treatment and stretching of treatment intervals occurred more often in 2022 (38.4%) than in 2021 (27.4%). 12 COVID-19-infections occurred overall, with typical only mild symptoms. The rate of fully vaccinated patients was 61.6% and 98.6% in May–July 2021 and 2022, respectively. Only minor side-effects after vaccination were reported.

**Conclusion:**

Despite mitigation of COVID-19 restrictions from 2021 to 2022, the health-care situation of patients worsened in this time. Reasons could be the international shortage of immunoglobulins during the pandemic and reduced physio/ergotherapy due to lingering regulatory restrictions. Vaccination rate was high in our cohort of patients compared to the general German population and CIDP did not seem to be a risk factor for severe SARS-CoV-2 infections.

## Introduction

SARS-CoV-2 pandemic has disrupted and continues to affect healthcare processes and the care of chronic patients until today. On March 11th 2020, the World Health Organization (WHO) declared the COVID-19 disease a pandemic. To date, a total of 578 million infections and 6.4 million deaths have been recorded worldwide (as of 02/08/22) [1]. This makes it the largest pandemic of the twenty-first century to date. Due to the rapidly changing situation on national treatment strategies and simultaneously intensive media coverage and discussion of scientific facts, with a non-scientific audience, at the beginning of the pandemic, the public was left with uncertainty, concern and fear of infection. Various individual medical and epidemiological measures were implemented to protect at-risk groups, as their course of disease was considered more severe and dangerous than of the normal population. These risk groups include individuals with autoimmune diseases with or without immunomodulatory therapy. This includes patients with chronic inflammatory neuropathies [2, 3].

Chronic inflammatory demyelinating polyneuropathy (CIDP) is the most common chronic inflammatory neuropathy and has a prevalence of 1–9 cases per 100,000 [4]. The pathomechanism of CIDP is a chronic demyelination of proximal and distal nerve segments. Cell-mediated and humoral immune responses are thought to be jointly responsible for the pathogenesis [5]. There are different therapeutic approaches depending on the subtype and treatment success. Intravenous (IVIG) or subcutaneous immunoglobulins, glucocorticoids intravenously or intrathecally, immunosuppressive drugs such as azathioprine, or as well as plasmapheresis are frequently used as treatment options [6].

Due to both the COVID-19 disease and the epidemiologic countermeasures of the SARS-CoV-2 pandemic, the health care system was put under a severe strain. Outpatient appointments and elective hospital admissions were canceled or postponed [7], and additional therapies such as occupational and physical therapy could not be provided on a regular basis because of the lockdown or staff shortages [7, 8]. It was suspected that the pandemic-related commodity crisis and worsening supply shortages of human immunoglobulin preparations [9, 10] could have a negative impact on patient care. In many cases, the requirement and interval of immunotherapy had to be reassessed on an individual basis and the personal risk of a SARS-CoV-2 infection had to be evaluated. Due to the underlying autoimmune disease, i.e. chronic inflammatory neuropathy, in our patients, they were classified as a high-risk group [2, 3] and their disease course was considered to be potentially more severe. With the advent of SARS-CoV-2 vaccines, it was necessary to evaluate the immune response to the vaccination in patients on immunomodulatory therapy. However, regarding chronic inflammatory neuropathies, it is unclear which vaccine and how many vaccinations will provide adequate protection against infection and what side effects might occur [11].

To provide better care for patients with chronic inflammatory neuropathies in future pandemics and during the current pandemic, we collected data on medical care during the SARS-CoV-2 pandemic from patients with chronic inflammatory neuropathies.

## Methods

A COVID-19 questionnaire was developed consisting of 11 questions with the following 5 subcategories: general care situation, change in immunotherapy, implementation of physical and occupational therapy, infection with SARS-CoV-2, vaccination, and vaccination side effects. The questionnaire consists mainly of multiple-choice questions, as well as free-text questions.

A total of 150 patients with CIDP received this questionnaire. The survey was conducted at two time points (May/June 2021 and May/June 2022). Patients were recruited from the INHIBIT registry (ethics vote 18-6534-BR dated June 12th 2019).

In addition, as part of the INHIBIT registry, the INCAT-Overall disability sum score (ODSS) was obtained from the patients surveyed through annual study examinations to assess the degree of disability [12].

We also analyzed how lockdown and contact restrictions affected our registry research, particularly nerve conduction studies (NCS) as a predictor of on-site study appointments. NCS are performed once a year as part of our INHIBIT register. It was temporarily not possible to conduct NCS or any other clinical examination and instead there was a data collection by phone. Therefore, we compared the number of telephone interviews without clinical examination with on-site study appointments including NCS beginning in January 2020 until June 2022.

CIDP and variants were diagnosed using EAN/PNS criteria 2021, multifocal motor neuropathy (MMN) was diagnosed according to EFNS/PNS criteria 2010 [13].

Data were analyzed using Excel (version 16.65, Microsoft, Redmond, WA, USA).

## Results

Of the 150 patients who received the questionnaire, 73 patients fully answered the questionnaire at both timepoints. These 73 patients were included in the study. Of these 34 had typical CIDP, 23 had distal CIDP, 10 had multifocal CIDP, 1 had a sensory CIDP, 4 had MMN, and 1 had a paranodopathy. Baseline disease characteristics of the patients are shown in Table 1.

**Table 1:** Baseline characteristics of study population

	Typical CIDP (*n* = 34)	Distal CIDP (*n* = 23)	Multifocal CIDP (MADSAM) (*n* = 10)	Sensory CIDP (*n* = 1)	MMN (*n* = 4)	(Para)-nodopathies (*n* = 1)
Gender, male/female	22/12	19/4	10/0	1/0	2/2	0/1
Age at examination, median, range	63.4 years (29–82)	64.1 years (49–80)	59.6 years (42–73)	56 years (56)	46.8 years (30–59)	31 years (31)
Disease duration from diagnosis until examination, median, range	2021: 64.3 months (0–276)	2021: 40.8 months (7–93)	2021: 56.2 months (7–152)	2021: 13 months	2021: 124 months (22–329)	2021: 32 months
	2022: 76.3 months (12–288)	2022: 52.8 months (19–105)	2022: 68.2 months (19–164)	2022: 25 months	2022: 136 months (24–341)	2022: 44 months
INCAT-ODSS at time of examination, median, range	2021: 4 (1–9)	2021: 2 (0–4)	2021: 4 (2–7)	2021: 2	2021: 2 (1–4)	2021: 0
	2022: 4 (1–7)	2022: 2 (0–6)	2022: 3 (1–9)	2022: 1	2022: 3 (1–4)	2022: 4
Number of patients receiving (therapy data based on 2022 data)						
IVIG	21	12	8		4	
SCIG	2	5	2			
Rituximab	4	4	4			
Azathioprine	4	4	1	1		1
Mycophenolate-mofetil	4	5				
Glucocorticoids	11	2				
Cyclophosphamide	1					
Ciclosporin-A	2					

*CIDP* Chronic inflammatory demyelinating polyneuropathy, *MADSAM* multifocal acquired demyelinating sensory and motor neuropathy, *MMN* multifocal motor neuropathy, *INCAT-ODSS* Inflammatory Neuropathy Cause and Treatment-Overall disability sum score, *IVIG* intravenous immunoglobulins, *SCIG* subcutaneous immunoglobulins

### Overall health care situation

**Fig. 1:**
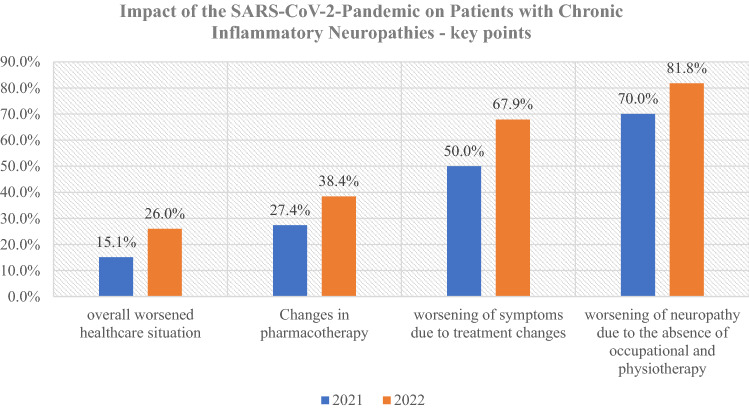
Impact of the SARS-CoV-2-pandemic—key points

In 2021, 15.1% of the patients in the cohort reported an overall worsened health-care situation. 26.0% reported this in 2022 (Fig. 1). In 2021, 17.9% did not keep to their medical appointments as before the pandemic, 16.3% reported this in 2022. 19.2% went to the physician less often because of the neuropathy in 2021, 13.7% in 2022. 17.8% had increased telephone contact with their treating physicians in 2021, 13.7% reported this in 2022.

### Changes in occupational and physiotherapy

Before the pandemic, 46.6% of patients regularly performed physiotherapy and occupational therapy was 43.8% in 2022. During the pandemic, in 2021, 29.4% reported the absence of physiotherapy and occupational therapy, and in 2022, the absence was reported 34.4%. Treatment interruptions varied between 1 and 52 weeks, median 18 weeks, in 2021. In 2022, the therapy was interrupted between 4 and 70 weeks, median 18 weeks. Patients reported a worsening of neuropathy due to the absence of occupational and physiotherapy in 70% in 2021 and in 81.8% in 2022 (Fig. 1).

### Changes in pharmacotherapy

Changes in treatment were reported by 27.4% (total *n* = 73) of the patients in 2021 and 38.4% in 2022 (Fig. 1). A change in treatment with immunoglobulins was the largest proportion of these. Regarding changes of treatment with immunoglobulins (*n* = 9) in 2021, the therapy was changed from subcutaneous immunoglobulins to intravenous immunoglobulins in one patient. One received lower dosage of intravenous immunoglobulin with the same interval. Treatment intervals of intravenous immunoglobulin changed in two patients. In three patients, the treatment intervals of subcutaneous immunoglobins changed.

Regarding changes of treatment with immunoglobulins (*n* = 22) in 2022, the therapy was changed from subcutaneous immunoglobulins to intravenous immunoglobulins in five patients. One received lower dosage of intravenous immunoglobulin with the same interval. Treatment intervals of intravenous immunoglobulin changed in 16 patients.

Due to these changes in therapy, 50% reported a worsening of symptoms in 2021 and 67.9% in 2022 (Fig. 1). Further information on the number of patients reporting a worsening of symptoms with changes in treatment are given in Table 2.

**Table 2:** Number of patients reporting a worsening of symptoms in relation to the number of patients who reported a change in treatment (in brackets). Change of treatment includes reduced dosage, longer interval or discontinuation

	2021	2022
Immunoglobulins	4 (9)	18 (22)
Corticosteroids	3 (5)	3 (3)
Rituximab	3 (5)	2 (2)
Other	1 (1)	1 (1)

### SARS-CoV-2 infections

Overall, 18 SARS-CoV-2 infections occurred in our study cohort. Most infections were in spring 2022 with the omicron variant (50% of the patients). All patients had typical but only mild symptoms, i.e., cold, coughing, sore throat, headache and aching limbs, fever and fatigue. Neither hospitalization nor ventilation was required in any case. 33.3% of the patients with an SARS-CoV-2 infection still reported ongoing symptoms like fatigue and concentration difficulties of their infection at the time of the survey in 2022. Due to isolation during the COVID-19-infection, three patients had to pause their current treatment. In two out of the three cases, pausing the treatment led to a stretching of the intravenous immunoglobulin intervals.

### SARS-CoV-2 vaccination

In the period of the first data collection 2021, the proportion of patients fully vaccinated was 61.6% (total *n* = 73). At the timepoint of our second data collection 2022, 98.6% were fully vaccinated (total *n* = 73). In May to July 2022 91.8% already got their booster-vaccination. 19.4% had also received their fourth vaccination.

The most first-time vaccinations were applied in April 2021 (48.6%). The second dose was mostly administered in May/June 2021 (68.1%). In December 2021, the most booster vaccinations were performed (41.8%).

In 2021, 68.9% of the first dosages have been vaccinated with Comirnaty^®^ (BioNTech), 26.2% with Vaxzevria^®^ (AstraZeneca) and 3.3% with Spikevax^®^ (Moderna). The second vaccination regimen was mostly Comirnaty^®^ (84.4% in 2021).

In 2022, 72.2% of the first dosages have been vaccinated with Comirnaty^®^, 22.2% with Vaxzevria^®^ and 4.2% with Spikevax^®^. The second vaccination was most commonly performed with Comirnaty^®^ (83.3% in 2022). 9.7% were vaccinated with Vaxzevria^®^ and 5.6% of the second dosages with Spikevax^®^. The booster vaccination was mostly performed with Comirnaty^®^ (64.2%) and Spikevax^®^ (35.8%) in 2022. 71.4% (total *n* = 14) of the fourth vaccinations in 2022 used Comirnaty^®^ and 21.4% Spikevax^®^.

Only minor side effects of the vaccinations were reported. Most common side effect was pain at the injection side (36.8% in 2021, 39.3% in 2022). The side effects lasted between 0.5 and 88 days, median 4.6 days in 2021 and 0.5 and 25 days, median 3.2 days in 2022.

One patient out of 72 has been infected with SARS-CoV-2 after the second dose and eight after the booster vaccination (data collected in the cohort of 2022). A total of nine patients reported a temporary mild worsening of tingling paresthesia after their second vaccination. None of the patients reported an objectifiable worsening of symptoms.

Table 3 gives an overview of results of the COVID-19 questionnaires.

**Table 3:** Excerpt of the results of the COVID-19 questionnaires

	*N* = 73	
	2021	2022
	%	%
Feeling of overall worsened health-care situation	15.1	26.0
Adjusted immunotherapy	27.4	38.4
Deterioration due to change in therapy	50.0	67.9
Medical appointments were attended as scheduled	82.1	83.7
Went to the doctor less often	19.2	13.7
Had more contact to the doctor by phone	17.8	13.7
Physiotherapy/occupational therapy		
Performed regularly as prior to pandemic	46.6	43.8
Had to interrupt during pandemic	29.4	34.4
Worsening due to interruption of therapy	70	81.8
COVID-19 infections	1.4	16.4
Omicron-variant	/	50
Hospitalization/Ventilation	0	0
Ongoing symptoms	100	33.3
Changes in therapy due to infection	0	25
Vaccination rate		
First vaccination	83.6	98.6
Second vaccination	61.6	98.6
Booster vaccination		91.8
Fourth vaccination		19.4
Vaccination with Comirnaty^®^		
First vaccination	68.9	72.2
Second vaccination	84.4	83.3
Booster vaccination	/	64.2
Fourth vaccination	/	71.4
Vaccination with Spikevax^®^		
First vaccination	3.3	4.2
Second vaccination	2.2	5.6
Booster vaccination	/	35.8
Fourth vaccination	/	21.4
Vaccination with Vaxzevria^®^		
First vaccination	26.2	22.2
Second vaccination	8.9	9.7
Booster vaccination	/	0
Fourth vaccination	/	0
Adverse vaccination side effects		
First vaccination	50.8	36.1
Second vaccination	57.8	41.7
Booster vaccination	/	29.9
Worsening of the polyneuropathy after vaccination		
After second vaccination	11.1	4.2
After booster vaccination	/	0

### Impact on register research

**Fig. 2:**
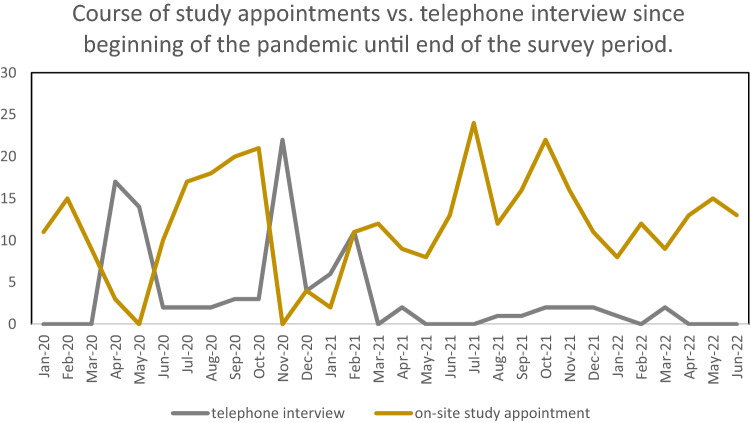
Study appointments vs. telephone interviews during the pandemic period

Figure 2 shows the number of telephone interviews without clinical examination compared to the on-site study appointments in our INHIBIT registry including NCS starting in January 2020 until the end of the survey period. There is a decrease in on-site study appointments at three time points: March to May 2020, October and November 2020, and January/February 2021. In the same time span, the number of telephone interviews had increased. From April 2021 the on-site follow-up examinations mostly continued as the pandemic progressed. At the same time, the number of telephone interviews is low.

## Discussion

Based on our evaluation, it is evident that the SARS-CoV-2 pandemic had a major and distinct impact on the medical care provided to our selected patient cohort of chronic inflammatory neuropathies. This is clearly different from the pandemic impact on the general German population.

Physician–patient contacts decreased, especially at the beginning of the pandemic, and stabilized at a low level by the time of our second survey. This could presumably be related to the fact that the health care system sectors did not have access to appropriate concepts for managing pandemics. Hospitals had to retain more capacity for COVID-19 patients, and elective procedures were postponed. Due to the additional workload and sick leave among clinic staff, there was an increase in staff shortages. Preventive measures to protect hospital staff and patients, such as mandatory testing, made it more difficult for patients to attend hospital and outpatient appointments. However, electrophysiological evaluation requiring direct physician–patient contact is essential since progressive axonal damage was shown even in asymptomatic limbs correlating with overall clinical disability [14, 15].

But contacts were also reduced on the patient side. People went to the hospital less often out of fear for potential infection [16–18]. This is also shown in the study of Musche et al. Despite evidence of reduced infections from immunotherapy in patients with CIDP and MMN, the prevalence of depression and fear of potential infection has increased [19].

In the second year of the pandemic, health system structures and processes adapted to the situation and strategies were deployed to continue providing patient care. In the second period of our survey, physician–patient contacts increased again, in time with the completion of the basic SARS-CoV-2 vaccination scheme (2 + 1 vaccinations being the standard at that time). Nevertheless, patients' subjective perceptions continued to deteriorate in 2021 15.1% felt to be in an overall worsened health care situation; in 2022 even 26.0%. Therefore, the frequency of physician–patient contact is not the only indicator of patients’ feeling of health care situation.

### Problems in physical and occupational therapy

Physical and occupational therapy could often no longer be provided as needed, due in part to staff sick leave, stricter hygiene regulations, and more restricted care [8]. This was also evident in our patient cohort. 29.4% (2021) and 34.4% (2022) were unable to continue their therapies as usual. This also led to worsening of CIDP symptoms in 81.8% in 2022. This confirms that physical and occupational therapy, or generally additional exercise therapy, is an important component in the treatment of neuropathies [20].

### Global shortage of intravenous immunoglobulins

Partially due to the global shortage of intravenous immunoglobulins, 27.4% (2021) of our patients required adjustments in therapy (38.4% in 2022) [9, 10, 18]. Despite the fact that the study of Romozzi et al. [21] indicates that patients were also able to cope with less or no immunoglobulins due to the pandemic, 50.0% (2021) of our cohort reported worsening of symptoms (67.9% in 2022). Due to the increased potential risk of infection in the clinic, it is also suggested by Romozzi et al. to discuss a switch to subcutaneous immunoglobulins, which could be self-administered by the patient in a home setting [21]. Due to supply shortages, this was not possible. Some patients even had to be switched back from subcutaneous immunoglobulins to intravenous therapy [9].

### COVID-19 disease

In our cohort, 12 patients were diagnosed with COVID-19. All patients experienced only mild courses. The risk for CIDP patients to contract COVID-19 is generally not higher than that of the general population [18, 22]. In our cohort, 16.3% of patients were infected with SARS-CoV-2 by June 2022, whereas approximately 33.6% of the German population were infected at the same point in time [23, 24]. Thus, the infection rate is significantly lower than the national average for the general population. This may be due to the fact that patients on immunomodulatory therapy have an increased risk of developing the disease, whereas patients on immunoglobulins did not show this risk increase [25]. In addition, a study by Stojanov et al. showed that patients with preexisting conditions, such as CIDP, limited their contacts more during the pandemic and stayed at home because they self-rated their risk for a severe course as increased [18].

### Vaccination data

Our cohort has an above-average vaccination rate compared to the average population. At the time of the first survey in June 2021, 98.6% of the cohort had already received basic vaccinations (two shots at a four-week interval). This compares to 36.5% basic immunization in the general population at that time-point [26]. By the time of the second survey, 91.8% in our cohort had received their booster vaccination. In comparison, in the general population aged 18 years and older, 71.9% had received their first booster vaccination at the same time [27]. This may be due to a higher willingness to vaccinate given the underlying disease and possible immunotherapies.

In eight cases in our cohort, worsening of symptoms of CIDP occurred after vaccination; however, symptoms resolved spontaneously in all cases. To date, there is a paucity of data on vaccination in patients with autoimmune neuritis. The Italian Peripheral Nervous System Association (ASNP) has not made a clear vaccination recommendation for CIDP patients due to this lack of information [11]. Thus, our data provides a valuable contribution for recommending COVID-19 vaccination for patients with chronic inflammatory neuropathies. This is also concluded by the study of Baars AE et al., which considered the COVID-19 vaccination as safe for said patients [28].

### Impact on register research

A significant decrease in the NCS can be seen in spring 2020 and autumn/winter 2020/2021, during the first lockdown in Germany from March 2020 until May 2020 and the second lockdown from November 2020 until March 2021 [29]. The on-site follow-up examinations mostly continued as the pandemic progressed.

It is fair to say that not only did patients experience a limited health care situation during the COVID-19 pandemic, but also research like our INHIBIT registry could not be continued as before

## Summary

Our data shows the impact of the SARS-CoV-2 pandemics on the health care situation of patients with chronic inflammatory neuropathies. Despite the mitigation of COVID-19 restrictions and recovering physician–patient contacts from 2021 to 2022, the overall health-care situation of patients even worsened during the pandemic. Our data shows treatment changes due to international shortage of immunoglobulins during the pandemic and reduced physio-/occupational therapy as probable reasons for this. Thus, there is a need to continue to work on coping strategies for this and future pandemics and to adopt new approaches to provide satisfactory care to patients.
